# The endocrine spectrum of Rathke cleft cysts

**DOI:** 10.3389/fendo.2025.1630695

**Published:** 2025-09-18

**Authors:** Natalia Rzewuska, Jacek Kunicki, Michał Kunicki

**Affiliations:** ^1^ Department of Gynecological Endocrinology, Medical University of Warsaw, Warsaw, Poland; ^2^ Department of Neurosurgery, Maria Sklodowska-Curie National Research Institute of Oncology, Warsaw, Poland; ^3^ INVICTA Fertility and Reproductive Center, Warsaw, Poland

**Keywords:** symptomatic Rathke cleft cyst, endocrine disorder, arginine vasopressin deficiency, menstrual disorders, pituitary tumors

## Abstract

Rathke cleft cysts (RCCs) are rare non-neoplastic lesions of the pituitary gland. Usually, these cysts are small and remain asymptomatic clinically. For unknown reasons, in some cases, RCCs enlarge and cause symptoms such as headaches, visual disturbances, and pituitary gland dysfunctions. The literature lacks comprehensive reviews or guidelines that summarize clinicians’ knowledge about hormonal assessment in symptomatic cases. We present a review of the literature focused on symptomatic cases of RCCs, manifesting with hormonal imbalance. Hormonal symptoms occur in 19.4–81% of symptomatic cases. The most common hormonal dysfunction is hyperprolactinemia, found in even 46% of cases, and the second most frequent is hypogonadism. The improvement after surgery is hesitant, between 19% and 67.8%, and is the worst in secondary hypothyroidism. In the pediatric patient group, hormonal dysfunctions are the most common presentation of such a lesion. Dysfunction of the posterior pituitary gland in the course of symptomatic RCCs can result in treatment-resistant arginine vasopressin deficiency and syndrome of inappropriate antidiuretic hormone secretion. It should be emphasized that among the endocrine disorders of RCCs in young premenopausal women, menstrual disorders and related fertility problems are prevalent. Irregular menstrual cycles or amenorrhea are reported in up to 17% of symptomatic RCCs. Endocrinologists and neurosurgeons must be acutely aware of hormonal imbalances in RCCs and conduct hormonal evaluations in every case of symptomatic RCC to enhance the management of these lesions. Guidelines for managing symptomatic cases of RCC are necessary to improve patient care and outcomes.

## Introduction

Rathke cleft cysts (RCCs) are common incidentalomas of the sella turcica region (1). The cysts are benign, asymptomatic lesions of the pituitary gland. In most cases, RCCs are accidentally found during images of the head for another reason. RCC was first described by Luschka in 1860, and the first symptomatic case was uncovered in 1913 (2). For some patients, RCC enlarges over time and causes symptoms such as headaches, visual impairments, endocrine dysfunctions, and arginine vasopressin deficiency (AVP-D) (3, 70).

Surgical treatment of RCCs, most commonly via the endoscopic transsphenoidal approach, is effective, but recurrence of this benign pathology occurs in 16-33% of cases, sometimes repeatedly, despite an adequately performed surgery (3, 4). Draining the contents of the cyst can potentially alleviate clinical symptoms, such as headaches and visual disturbances (5, 6). It is assumed that lesions should be observed for 5 years after surgery (5–7).

To date, no treatment guidelines have been developed for symptomatic cysts, and endocrine disorders are described as observational studies, usually as an experience from single centers (2, 8, 9).

The literature lacks review data on endocrine disorders in symptomatic cases, which would be helpful to clinicians in a practical way. There is no summary of reports regarding the time of observation, and treatment of cyst with hormonal disorders, including the decision of surgery in symptomatic Rathke cleft cysts (3, 10).

This study is a narrative review summarizing the literature on endocrine dysfunctions associated with symptomatic RCCs. The goal is to identify common clinical observations, assess current management strategies, and offer recommendations for clinical practice. Additionally, the review highlights research gaps and suggests future directions to improve the understanding and treatment of RCCs.

## Methods

This narrative literature review used PubMed, Embase databases, and the Google Scholar search engine. We looked for articles in English from January 1994 to January 2025, for a reason to investigate the problem described in the literature over 30 years. The study included cross-sectional and cohort studies, systematic review data, and case reports, with a significant description of endocrine dysfunction. We searched for analyses and reports on endocrine disorders in patients with symptomatic RCCs. Reviewers R.N. and J.K. collaborated closely to gather data from the full-text articles. They also consulted another reviewer, K.M., for guidance and input throughout the data extraction process. The keywords used for the literature search included: “Rathke cleft cyst,” “Pituitary cyst,” “Sellar cystic lesions,” “Non-neoplastic pituitary lesion,” “Pituitary dysfunction”, and particular hormonal imbalances: “Hyperprolactinemia,” “Hypogonadism”, “Secondary adrenal insufficiency”, “Diabetes insipidus”, “SIADH” and “Endocrine evaluation.” The primary outcome was the type and characteristics of endocrine disturbances in the course of symptomatic cyst. All authors discussed the results of the obtained information, including specific endocrine disorders, the management of these disorders, and the controversies surrounding them. The review summarizes the endocrine manifestations of RCC and offers management strategies given these symptomatic lesions.

## Discussion

RCCs, also known as part intermedia cysts, are benign, non-neoplastic lesions of the sella turcica (1). The symptomatic RCC constitutes 5 to 15% of the lesions in the stellar area (6). The prevalence of these lesions in routine autopsy studies is similarly notably high, reported in approximately 13–22% of cases (5). RCCs are a common incidentaloma of the sellar area, and in a single-center study, was constituted 42% among non-adenomatous lesions (11). A commonly encountered diagnostic error involves interpreting an incidentally detected intrasellar lesion as a pituitary adenoma on T1 and contrast-enhanced T1-weighted images, whereas it is actually a Rathke cleft cyst (12).

RCC originates from remnants of Rathke’s pouch, an embryonic structure situated in the region of the pars intermedia of the pituitary gland; however, the pathogenesis is not fully understood (2). Experimental research suggests that mutations or dysfunctions in the Isl1 transcription factor gene may play a role in the pathogenesis of the cysts (3). Pituitary development is regulated by signaling molecules from adjacent tissues and homeodomain transcription factors. Their interaction triggers the formation of Rathke’s pouch—an ectodermal invagination from the primitive oral cavity that emerges during the third to fourth week of gestation. In the later development it detaches from the pharynx and gives rise to the anterior pituitary. Proliferation of anterior wall cells forms the pars distalis and pars tuberalis, while the posterior wall develops into the pars intermedia. These structures differentiate into five hormone-secreting cell types. Remnants of Rathke’s pouch may persist as cysts lined by typically ciliated cuboidal or columnar epithelium (13).

**Figure 1:**
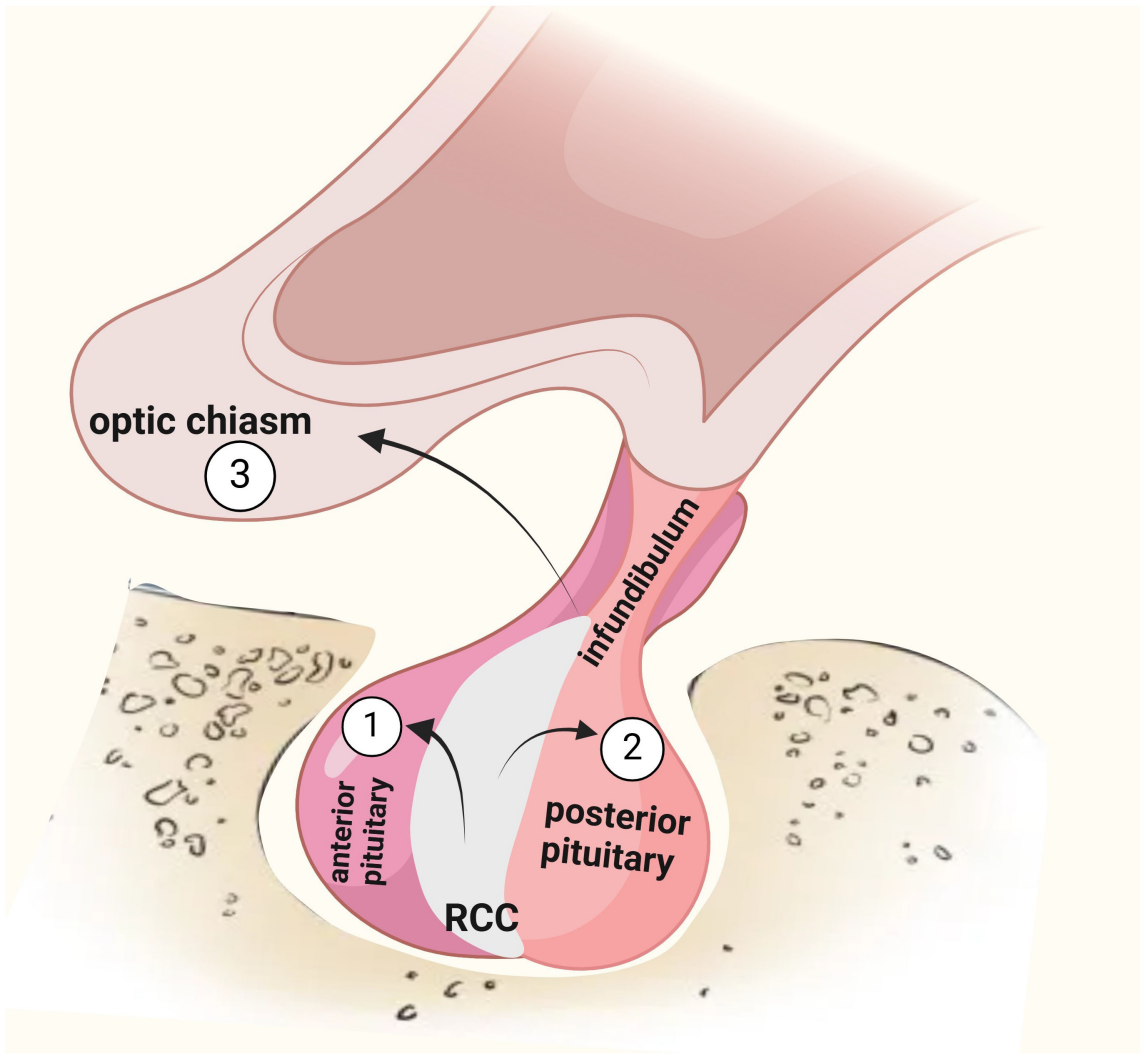
Symptoms arising from RCCs can be attributed to specific mechanisms. Within the sella, compression and the inflammatory process of the anterior pituitary gland can lead to hypopituitarism (1). Concurrently, effects on the posterior pituitary gland may result in arginine vasopressin deficiency (2). A suprasellar extension may exert pressure on the optic chiasm, leading to visual disturbances (3). These pathophysiological effects emphasize the importance of timely diagnosis and management.

RCCs are most commonly found in adults and primarily occur in individuals between 30 and 50 (5), showing the highest incidence within this age range and being more prevalent among females, with a predominance of 2:1 (9, 14). Cysts are usually small and are often accidentally discovered during head imaging for different reasons. Typically asymptomatic, they are found in about one in six healthy individuals undergoing brain imaging studies (1). Additionally, in the pediatric population, RCCs, along with craniopharyngioma, are the lesions most frequently encountered in the sellar region. Usually, these cysts are small (< 5 mm) and symptom-free. Still, as the cyst grows, it exerts a mass effect, leading to clinical symptoms such as headaches, visual impairments caused by optic chiasm compression, hypopituitarism, and, less commonly, AVP-D (**Figure 1**). However, the development of the hormonal disorder is more likely caused by the inflammation spreading to adjacent pituitary lobes than by mass effect (3, 4, 15, 16). The involvement of the cyst within the sella turcica suggests indirectly that the inflammatory process extends to the adjacent location (17). Acute onset of symptoms may also result from bleeding from the rupture of a large cyst. Repeated minor ruptures may progressively lead to irreversible damage to the hypophysis (16, 18).

## Hormonal presentation

Hormonal imbalances associated with the cyst include hyperprolactinemia, gonadal insufficiency, adrenal insufficiency (AI), and disruptions in the hypothalamic-pituitary-thyroid axis. The most effective approach to treating associated endocrinopathy has not yet been determined. Surgical intervention is typically chosen to address symptoms such as headaches and visual impairments; however, it is considered less effective for treating associated endocrinopathies and may carry a risk of additional complications (2). There is also limited research examining pituitary function in patients with RCCs who have not undergone surgical intervention. Earlier comprehensive studies identified endocrine dysfunction in approximately 19.4–81% of patients with cysts. Hormonal disorders documenting more than two hormonal axes dysfunction in different publications range from 10% to 59% (2, 16, 19–24). Another possible scenario is a deficit in more than two hypothalamic-pituitary axes, which is called panhypopituitarism (25). However, endocrine disorders may be largely subclinical, which could deteriorate the diagnosis (16). The most frequently reported abnormalities were hyperprolactinemia and hypogonadism. Hyperprolactinemia is considered the most common endocrine disorder and is estimated to be between 5% and 46%. Meanwhile, hypogonadism ranges from 3% to 60% of patients, depending on the study. Secondary AI is observed in 7.4% to 40% of cases. Interestingly, the experience of the two centers indicated that AI was the most common endocrine manifestation (3, 26, 27). The underlying mechanisms by which RCCs contribute to the development of Cushing’s disease remain unclear. In the available cases described in the literature, the diagnosis was based on histopathology, and the hypercortisolism was resolved postoperatively. In individuals present with clinical and biochemical evidence of Cushing’s disease alongside imaging that indicates a pituitary abnormality, RCC should be included in the differential diagnosis, even if no adenoma is identified. Further investigation is necessary to clarify whether RCCs have a direct impact on hormonal activity or stimulate increased secretion in adjacent pituitary cells (27).

In turn, growth hormone (GH) deficiency varies from 0 to 81% among different data sources. Additionally, hypothyroidism was observed in 4–44% of patients. In some previously described studies, no association was found between the pituitary gland size and pituitary dysfunction (2, 28). However, endocrine disorders were more frequently observed in the presence of a contrast-enhancing edge on Magnetic Resonance Imaging (16).

## Pediatric population

RCCs are observed less frequently in the pediatric population than in adults (29). Along with headaches, the most common symptom is mild endocrine disorders found in laboratory testing (30). In a single center’s experience, RCC in a group of children caused endocrine dysfunction in 59.3% of cases. Endocrine dysfunction was observed even more often among younger children (31). The findings from another center also showed a similar rate of endocrine disorders, with 61% affected. The conditions observed included central precocious puberty in 18% of cases, DI in 14%, general fatigue in 11%, and reduced growth velocity in 7% (17, 32).

In general, the most common initial symptoms of RCC are endocrine dysfunctions, such as impaired growth hormone secretion, elevated PRL levels, or AVP-D (33). Previous studies showed that growth impairment is one of the most common symptoms in the pediatric population. It is assumed that the ongoing inflammation caused by the cyst may affect the somatotropic cells (29). In patients with central precocious puberty, RCCs were sometimes found, but it is not clear whether it was the direct cause of such a condition. In turn, it is believed that the ongoing inflammation due to the cyst may affect the inhibitory pathways of the gonadotropin-releasing hormone (33). Hormonal laboratory tests can confirm abnormalities that match clinical signs and symptoms when they are present. Additionally, these tests may reveal subclinical issues such as mild hyperprolactinemia, hypocortisolism, hypogonadism, or hypothyroidism, which might not be evident during a clinical evaluation (30). Pediatric patients diagnosed with RCC need comprehensive clinical and hormonal evaluations, along with regular follow-up, to monitor the risks associated with hypopituitarism, the presence of coexisting pituitary tumors, and the potential for cyst enlargement (34). Once again, every cystic lesion within the sella turcica patient should undergo a thorough endocrine evaluation (9).

## Arginine vasopressin deficiency and SIADH

RCCs are rarely present with symptoms such as DI. This is reported in up to 0-67% of patients diagnosed with symptomatic cases (14, 23, 28, 35). The diagnosis is established by clinical symptoms, including polydipsia, polyuria, and excretion of over 2500 ml of urine during 24h. Laboratory tests include low levels of urine osmolality and low urine gravity. DI often remains persistent following surgery (36). The pathogenesis of DI remains unclear. One potential pathomechanism is the spread of inflammation resulting from a cyst rupture. DI may be linked to advanced stages of inflammation, transitioning from adeno-hypophysitis to infundibulo-panhypophysitis as the inflammatory process advances. DI is divided into two groups: acute and chronic types. Surgical treatment can resolve acute DI, but not the chronic one.

The syndrome of inappropriate diuresis (SIADH) is defined by euvolemic hyponatremia, typically presenting with low serum osmolality, elevated urine osmolality, and also increased urinary sodium concentration (37). Hyponatremia caused by SIADH is a very infrequent manifestation of RCC (38, 39). SIADH occurred preoperatively in up to 6% of a single-center study (35). The possible explanation for this causative situation is that RCC exerts pressure on the posterior pituitary, potentially triggering SIADH by releasing stored antidiuretic hormone into the bloodstream. Patients often exhibit nonspecific symptoms such as nausea, vomiting, headaches, confusion, seizures, and muscle cramps. They can vary in severity depending on the degree and rate of hyponatremia onset. In SIADH, antidiuretic hormone is inappropriately secreted under normal physiological conditions, leading to impaired water excretion and the development of dilutional hyponatremia. In cases where symptoms are severe or if complications such as SIADH occur, surgical resection of the cyst may be necessary (37).

Previous studies have shown that postoperative recovery from DI was achieved in only a tiny percentage of patients. Moreover, iatrogenic DI has been identified as a common complication, accounting for 4-19% of these cases (2, 5, 14). Also, SIADH is estimated to occur in about 13% postoperatively (20). In the surgical management of suprasellar RCCs, preserving the pituitary stalk and hypothalamus should precede aggressive cyst resection to minimize potential complications and ensure better functional outcomes (40).

## Menstrual and gonadal disorders

The prevalence of impaired sexual function caused by RCC has been reported to range from 8% to 58% of symptomatic cases (9, 23, 41, 42). It is well known that a regular menstrual cycle reflects good health status and perception of well-being and indicates the appropriately acting hypothalamus-pituitary-ovarian axis (43). Pituitary tumors, which could be hormonally active adenomas or nonactive, can lead to menstrual cycle disturbances and infertility. The pituitary adenomas can release PRL, GH, and other hormones. Both hormonally active and inactive can sometimes cause mass effects and disrupt the hypothalamic-hypophysis-ovarian axis. In turn, that can lead to hypogonadotropic hypogonadism and, as a result, oligomenorrhoea, amenorrhea, and infertility (43).

Primary amenorrhea is when a woman has never had a menstrual period and is 16 years old or older with signs of secondary sex characteristics, or 14 years old if there is no sign of secondary sex characteristics. Secondary amenorrhea is defined as the absence of menses for >3 months in girls or women who previously had regular menstrual cycles or 6 months in girls or women who had irregular menses (44, 45).

Certain studies suggest a higher prevalence of RCCs in females, likely due to earlier detection associated with menstrual irregularities or amenorrhea caused by gonadotropin deficiency (27).

However, RCCs rarely cause amenorrhea and infertility. Menstrual cycle irregularities were observed in 7% to 24% of women with this lesion (3, 9, 16). The data usually comes from a few sources, mainly case reports. In a case report on an Asian population, they described, for the first time, an infertile 32-year-old woman with RCC presenting with secondary amenorrhea and infertility. The amenorrhea developed after her first spontaneous delivery when she was 20 years old. The patient’s menstruation was regular before her first pregnancy. After some years of amenorrhea, she was diagnosed with RCC. During the laboratory assessment, she had normal anti-Mullerian hormone serum levels and thyroid-stimulating hormone. The patient’s menstruation was regular before her first pregnancy; however, she manifested amenorrhea after her first spontaneous delivery in her twenties. Unlike FSH and LH, PRL and estradiol were below the normal range. Additionally, in sonography findings, she presented atrophied ovaries and uterus. She was successfully treated with the third cycle of ovulation induction using human menopausal gonadotropin and human chorionic gonadotropin, and finally got pregnant (44). Authors speculated that amenorrhea and infertility have been caused by inflammatory reactions in the cyst wall and adjacent tissue that result in pituitary destruction and pituitary function (46).

In another retrospective study of 73 cohort women with RCC, amenorrhea was found in 16% of cases. The median age of the group was 43 years, and 45/73 (62%) women were premenopausal. In two cases, the women were also diagnosed as having polycystic ovarian syndrome. The authors also did not find a correlation between the cyst size and pituitary function. Interestingly, half of the women were overweight or obese (47).

The retrospective study by Eguchi et al. found menstrual disturbances in 3 out of 9 women. All these women were of reproductive age, from 21 to 35 years. The galactorrhea also occurred in 2 of them. According to the authors, amenorrhea is a common symptom in female patients with RCC. They speculate that the mechanism leading to the cessation of menses could be complex. It compromises both hyperprolactinemia due to the cyst’s pituitary stalk compression and impaired gonadotropin secretion. Additionally, because not only intrasellar lesions can cause amenorrhea, the inflammation process could be taken into account (19). In a single-center experience, more than 65% of patients with RCC experienced improvements in symptoms such as amenorrhea, galactorrhea, and oligomenorrhea postoperatively. In those groups of patients, the most common endocrine symptom was decreased libido and impotence among men. This proves that clinical symptoms related to gonadotropin secretion should be mainly considered in the case of hormonal evaluation of cysts (14). It is important to remember that in the context of gonadal disorders, men may experience clinical symptoms such as sexual dysfunction and reduced libido (3, 9, 25).

## Hormonal diagnostics

Clinical and hormonal assessments should be conducted for every pituitary lesion, and the best with cooperation with an endocrinologist (9, 48, 49). The endocrine evaluation should be performed both pre- and post-operatively, according to the Endocrine Society recommendations (50). The assessment should include baseline insulin-like growth factor-1 (IGF-1) levels, adrenal, thyroid, and gonadal function assessments, and PRL levels. Hyperprolactinemia is identified as PRL levels exceeding the normal range for the patient’s sex (26). Central AI should be diagnosed based on an abnormal cortisol response to a 1 µg adrenocorticotropic hormone stimulation test (30-minute stimulated cortisol <16 µg/dL or 16–18 µg/dL with clinical signs and symptoms of AI). Central hypothyroidism is characterized by low free thyroxine levels along with low or normal thyroid-stimulating hormone levels. In men, central hypogonadism signifies low testosterone levels along with low or normal follicle-stimulating hormone (FSH) and luteinizing hormone (LH). For premenopausal women, the diagnosis is based on oligo or amenorrhea, low estradiol, and low or normal FSH and LH levels. In turn, in postmenopausal women, central hypogonadism is specified as FSH and LH levels below the postmenopausal reference range. Multiple hormone deficiencies are characterized by more than one hormonal deficiency, including IGF-1 levels below the age- and sex-adjusted reference range. GH stimulation tests are typically not conducted in patients with multiple pituitary deficiencies since low IGF-1 levels suggest GH deficiency, making stimulation tests optional (26, 51). Central AVP-D (DI) should be diagnosed according to clinical symptoms such as excessive urination (polyuria) and increased thirst (polydipsia) combined with an inappropriately low urine osmolality. These findings highlight the critical importance of assessing IGF-1, hormonal levels, and adrenal reserve in patients with RCC, regardless of the cyst’s size (26). Regular Magnetic Resonance Imaging monitoring and endocrine assessments are advised, as most recurrences are observed within the first five years following surgery (52).

## Differential diagnosis

The main conditions considered in the differential diagnosis of Rathke’s cleft cysts include craniopharyngiomas, cystic pituitary adenomas, and arachnoid cysts (1).

A common misdiagnosis is identifying an RCC as a pituitary adenoma based on imaging studies, usually only on T1 and T1 with contrast-enhanced images. Intrasellar cysts larger than 5 mm typically appear oval, are vertically oriented, and are positioned along the midline, anterior to the posterior pituitary lobe. Coronal T2-weighted imaging helps differentiate RCC from other pituitary lesions by showing the cyst surrounded bilaterally by the pituitary gland — a pattern not seen in Pituitary Neuroendocrine Tumors. Additionally, about half of RCCs show a characteristic hypointense intracystic nodule on T2-weighted images. Another common error is confusing the posterior pituitary bright spot with a mucoid RCC on sagittal T1-weighted images, which can be clarified with T2-weighted or axial T1 sequences (12).

In turn, the collision of the RCC with other pituitary tumors is a rare phenomenon and a diagnostic challenge. Several cases have been reported where the cyst coexists with a pituitary adenoma. The knowledge about tumor collisions is based on case reports (53, 54). Immunohistochemical findings suggest that most of these adenomas are prolactin-secreting. Fewer involve growth hormone-secreting, corticotrophic, or nonfunctioning adenomas. There have also been descriptions of complex sellar lesions composed of pituitary adenomas closely associated with RCC and areas of metaplastic squamous epithelium. It remains uncertain whether these represent true transitional tumors or are simply the result of a close spatial association between a cyst and an adenoma (53).

Collision lesions, such as a microprolactinoma occurring alongside a cyst, can complicate the diagnosis. In such a case, it is good to remember that prolactinomas respond rapidly to treatment with dopamine agonists, often within just a few days, showing heightened T2-weighted hyperintensity on pituitary MRI. Additionally, the cavernous sinus compartment also decreases in size. They also push the pituitary stalk forward. In the context of a cystic sellar mass, hyperprolactinemia requires cautious interpretation, as a prolactin concentration around 100 μg/L may reflect either a cystic macroprolactinoma with minimal solid tissue or hyperprolactinemia secondary to a stalk effect, with a potential mismatch between lesion size and prolactin levels (12). Notably, in cases of collision lesions, the microadenoma both displaces and deforms the Rathke cleft cyst, due to the cyst’s low internal pressure (55). RCCs have also been documented in association with adenomas that produce ACTH and growth hormone (56). ACTH-producing adenomas causing Cushing Syndrome are an infrequent situation. Inferior Petrosal Sinus Sampling (IPSS) is the gold standard for distinguishing ectopic from central Cushing’s syndrome; however, some studies have shown that it may yield false-negative results in cases of cyclical Cushing’s disease (54). Since unrecognized and untreated subclinical hypopituitarism can negatively impact sexual health, bone density, and cardiovascular function, it is essential to assess the hypothalamic–pituitary axis whenever a pituitary mass is identified (56).

One of the greatest challenges in diagnosing a Rathke’s cleft cyst (RCC) lies in distinguishing it from a craniopharyngioma (**Table 1**). Rathke’s pouch remnants often give rise to small cystic formations, which, while sometimes present in children, occur most frequently in adults. RCCs that exhibit pronounced squamous metaplasia can closely resemble papillary craniopharyngiomas in their macroscopic appearance, imaging features, and microscopic characteristics (1). RCC is usually lined with columnar or squamous epithelial cells, sometimes with cilia (14). In the histopathological picture, there may be some secondary changes, including squamous metaplasia, characteristic of inflammation and hemorrhage. The presence of squamous metaplasia increased the risk of papillary craniopharyngioma (57).

**Table 1:** Comparison of Rathke’s cleft cyst and craniopharyngioma (CP) (1, 57).

Feature	Rathke’s Cleft Cyst	Papillary Craniopharyngioma
Origin	Remnant of Rathke’s pouch	Neoplastic lesion, BRAF V600E mutation-driven
Histology	Cyst wall lined by cuboidal/columnar epithelium, with occasional squamous metaplasia	Squamous epithelium with papillary architecture
Molecular Marker	Typically BRAF V600E negative	BRAF V600E positive in nearly all cases
Clinical Presentation	Often incidental; can cause headaches, visual disturbances, and pituitary dysfunction	Similar symptoms, but often more aggressive and recurrent
Radiologic Features	Cystic sellar/suprasellar lesion; no calcifications	May mimic RCC radiologically, lacks the classical calcifications of adamantinomatous CP
Diagnosis Challenges	May be difficult to distinguish from pCP histologically	Can be misdiagnosed as RCC without molecular testing
Recurrence Rate	Lower; recurrence possible but less aggressive	Higher, often requires multiple interventions
Main Management Strategy	Observation or surgical drainage	Surgery ± possible BRAF inhibitor therapy
Recommended Additional Testing	BRAF testing not standard but suggested in cases with recurrence or squamous metaplasia	BRAF testing recommended and confirms diagnosis

## Management

When the cyst increases in size and/or causes symptoms, surgical intervention is typically advised. Available techniques range from simple cyst drainage (with or without fat graft placement) to marsupialization, extensive removal of the cyst wall, or even irrigation with a caustic agent such as alcohol (58). Conversely, complete removal of the lesion carries the risk of transient or permanent complications arising from manipulation within the intricate anatomical region surrounding the cyst. Potential complications include AVP-D, long-term pituitary insufficiency, visual impairments, vascular injury, and cerebrospinal fluid leakage (5, 6). Presently, there are no established guidelines specifying when radical resection involving cyst wall removal should be performed versus employing cyst aspiration and drainage alone to minimize complications (5–7). Detection of residual cysts after surgery is associated with a greater chance of recurrence (27).

Besides surgical approaches, both stereotactic radiosurgery (SRS) and fractionated stereotactic radiotherapy (SRT) have proven to be effective options for treating recurrent RCC. These treatments are directed at the cyst wall, which contains the cells that produce the cyst’s contents. By targeting this area, the approach is thought to decrease the production of cyst material and thereby lower the likelihood of recurrence (58). In a series of cases involving multiple recurrences of the cyst initially managed with surgical drainage, followed by salvage treatment using fractionated stereotactic radiotherapy. The patients achieved sustained disease control along with favorable neurological and endocrine outcomes. In rare but difficult-to-manage cases, fractionated stereotactic radiotherapy offers a safe, practical, and effective salvage option (59). Based on the available studies, it can be concluded that stereotactic radiotherapy has low side effects, but among them it is necessary to mention new endocrinopathies, including those affecting the adrenal and thyroid axes. Radiation therapy may be an appropriate option for patients who are poor candidates for surgery or have had multiple recurrences following previous surgical interventions (58). The management of RCC includes surgical treatment or close observation. Asymptomatic cases with cyst diameter less than 1 cm, without visual examination and pituitary function, can be managed conservatively with serial MRIs. Clinically stable cases should undergo clinical evaluation with visual functioning testing yearly, with MRI follow-up at 1, 3, and 5 years (1, 60). The 5-year observation period seems to be the most accurate, due to the previous reports about the stable diameter of the cyst in patients with a baseline diameter less than 1 cm (27). Nevertheless, the some recent studies emphasize the importance of a minimum 6-year follow-up, only a few reports have extended their observation beyond that period (61).

## Postoperative improvement

The improvement in hormonal disorders after cyst surgery ranges from 19% to 67.8% across different studies (2, 3, 14, 62). The results of the surgical effect may depend on the surgical treatment method, and minimally invasive transsphenoidal surgery at a single center had the best treatment effects. Despite surgical treatment, a lack of improvement in hypopituitarism can be expected in 20% of patients on average (2, 5). Additionally, microscopic transsphenoidal surgery seems to have a higher rate of iatrogenic endocrine dysfunction compared to using an endoscope technique (63).

Unfortunately, surgical treatment may also deteriorate the pituitary gland function, and as many as 4-30% will have worsening endocrine symptoms (3, 9, 10, 14, 20, 26, 64). In addition, symptom improvement can be expected depending on the type of symptoms. Some data indicate that more impressive improvement, besides hyperprolactinemia, is seen in GH deficiency or hypogonadism. The worst potential for improvement lies in hypothyroidism (**Table 2**) (23, 62).

**Table 2:** The incidence of different endocrine dysfunctions (3, 14, 26, 27, 65) among various studies and postoperative improvements (3, 10, 25, 26, 64, 65).

Type of hormonal dysfunction	Preoperative incidence [%]	Postoperative improvement [%]	Associated cyst features
Hyperprolactinemia	5–46%	Up to 100%	More likely with stalk effect; often improves after decompression (64, 65)
Growth Hormone (GH) Deficiency	0–81%	18–33.3%	Frequently associated with suprasellar extension; often persistent if longstanding (3)
Hypogonadism	3–60%	18–30%	May correlate with larger or suprasellar cysts; partial recovery possible (3)
Secondary Hypothyroidism	4–44%	0–44.4%	Common in intrasellar cysts; variable recovery postoperatively (3)
Secondary Adrenal Insufficiency	7.4–40%	14–56.3%	More frequent in intrasellar lesions; recovery depends on timing of intervention (3, 65)
Multiple Hormonal Axis Deficits	10–59%	~16.7%	Often seen in larger cysts or longer-standing cases (64, 65)
General Pituitary Dysfunction	19–67.8%	Up to 50% in general and 56.2% improvement in anterior pituitary axis	Linked to larger cyst size, suprasellar extension, and mass effect on the pituitary. Findings consistent across multiple retrospective studies; dysfunction is more likely in RCCs with mass effect or unfavorable anatomical positioning (10, 65)

In the case of AI, a single occurrence of this symptom should not be an indication of the surgery due to poor surgical success in improving such a symptom (26). As mentioned, hormonal disorders are probably caused by the inflammation that spreads to the adjacent pituitary gland. It is believed that the duration of the chronic condition may impact the deterioration of the pituitary function. Therefore, in older patients, where the process could take place for an extended period, a worse recovery of the pituitary function was observed compared to the younger patient group (2). It is worth underlining that the coexistence with pituitary adenoma, which is estimated at 11%, can lead to pituitary dysfunction, mainly by increased GH secretion and hyperprolactinemia. Some studies showed that early surgical intervention in patients with hypopituitarism may help with the coming progressive dissipation (36). Although other papers suggest that preoperative hypopituitarism may improve in approximately one-third of cases, the other literature shows a minimal impact on restoring pituitary function. The notable exception is the “stalk effect,” which causes hyperprolactinemia. In this case, excess prolactin (PRL) following the surgical decompression resolves in almost every case (20, 62, 66). The observed postoperative decrease in PRL levels could result from reestablishing dopamine-mediated inhibition from the hypothalamus or potentially reflect impaired function of the anterior pituitary gland (14).

We emphasize the need for comprehensive hormonal evaluation in patients with symptomatic RCCs (52). The Endocrine Society recommended that all pituitary axes should be re-evaluated beginning six weeks after pituitary surgery. Regular follow-ups should be conducted to track the emergence or resolution of pituitary dysfunctions. The decision about surgery should be individualized for each case of pituitary dysfunction caused by a pituitary tumor (50, 52).

## Conclusions

An adequate summary of the endocrine symptoms has not been found among the many publications on RCCs in medical databases. There are also no guidelines regarding surgical indications or the timing of the surgery itself. Prompt surgical intervention for RCC accompanied by hypopituitarism may help halt the progression of hypopituitarism (67). Collaboration between neurosurgeons, endocrinologists, and radiologists is essential to optimize treatment strategies and ensure patient outcomes (37, 68). RCCs require thorough clinical and hormonal evaluations, as well as ongoing monitoring, due to the risks of hypopituitarism, coexisting pituitary tumors, and potential cyst enlargement (34). Every cystic lesion in the sella turcica patient should undergo a comprehensive endocrine evaluation to ensure accurate diagnosis and appropriate management (50, 69). Surgical treatment should be contemplated when an RCC is suspected in the presence of endocrine-related symptoms (56).

## References

[1] Hacioglu A Tekiner H Altinoz MA Ekinci G Bonneville JF Yaltirik K. Rathke’s cleft cyst: From history to molecular genetics. Rev Endocr Metab Disord. (2025) 26:229–60. doi: 10.1007/s11154-025-09949-6, PMID: 39939491 PMC11920404

[2] Park JK Lee EJ Kim SH. Optimal surgical approaches for Rathke cleft cyst with consideration of endocrine function. Neurosurgery. (2012) 70:250–6. doi: 10.1227/NEU.0b013e3182418034, PMID: 22089758

[3] Kim E. Symptomatic Rathke cleft cyst: clinical features and surgical outcomes. World Neurosurg. (2012) 78:527–34. doi: 10.1016/j.wneu.2011.12.091, PMID: 22381268

[4] Han SJ Rolston JD Jahangiri A Aghi MK. Rathke’s cleft cysts: review of natural history and surgical outcomes. J Neurooncol. (2014) 117:197–203. doi: 10.1007/s11060-013-1272-6, PMID: 24146189

[5] Aho CJ Liu C Zelman V Couldwell WT Weiss MH. Surgical outcomes in 118 patients with Rathke cleft cysts. J Neurosurg. (2005) 102:189–93. doi: 10.3171/jns.2005.102.2.0189, PMID: 15739543

[6] Altuwaijri N Cote DJ Lamba N Albenayan W Ren SP Zaghloul I. Headache resolution after rathke cleft cyst resection: A meta-analysis. World Neurosurg. (2018) 111:e764–e72. doi: 10.1016/j.wneu.2017.12.170, PMID: 29309984

[7] Fukui I Hayashi Y Kita D Sasagawa Y Oishi M Tachibana O. Significant improvement in chronic persistent headaches caused by small rathke cleft cysts after transsphenoidal surgery. World Neurosurg. (2017) 99:362–8. doi: 10.1016/j.wneu.2016.12.111, PMID: 28057594

[8] Komatsu F Tsugu H Komatsu M Sakamoto S Oshiro S Fukushima T. Clinicopathological characteristics in patients presenting with acute onset of symptoms caused by Rathke’s cleft cysts. Acta Neurochir (Wien). (2010) 152:1673–8. doi: 10.1007/s00701-010-0687-5, PMID: 20495985

[9] Dadej D Skraba K Matyjaszek-Matuszek B Świrska J Ruchała M Ziemnicka K. Presenting symptoms and endocrine dysfunction in Rathke cleft cysts - a two-centre experience. Endokrynol Pol. (2021) 72:505–11. doi: 10.5603/EP.a2021.0091, PMID: 34855191

[10] Nakase K Nishimura F Yokoyama S Kakutani M Morisaki Y Kotsugi M. Long-term outcomes and potential predictive recurrence factors after endonasal endoscopic surgical treatment of symptomatic Rathke’s cleft cysts. Neurosurg Rev. (2024) 47:85. doi: 10.1007/s10143-024-02322-2, PMID: 38366128

[11] Valassi E Biller BM Klibanski A Swearingen B. Clinical features of nonpituitary sellar lesions in a large surgical series. Clin Endocrinol (Oxf). (2010) 73:798–807. doi: 10.1111/j.1365-2265.2010.03881.x, PMID: 20874772 PMC2982869

[12] Bonneville J-F. Tricks and Traps in MRI of the Pituitary Region. Cham: Springer (2024). p. 244.

[13] Cushman LJ Watkins-Chow DE Brinkmeier ML Raetzman LT Radak AL Lloyd RV. Persistent Prop1 expression delays gonadotrope differentiation and enhances pituitary tumor susceptibility. Hum Mol Genet. (2001) 10:1141–53. doi: 10.1093/hmg/10.11.1141, PMID: 11371507

[14] Shin JL Asa SL Woodhouse LJ Smyth HS Ezzat S. Cystic lesions of the pituitary: clinicopathological features distinguishing craniopharyngioma, Rathke’s cleft cyst, and arachnoid cyst. J Clin Endocrinol Metab. (1999) 84:3972–82. doi: 10.1210/jcem.84.11.6114, PMID: 10566636

[15] Nishioka H Haraoka J Izawa H Ikeda Y. Magnetic resonance imaging, clinical manifestations, and management of Rathke’s cleft cyst. Clin Endocrinol (Oxf). (2006) 64:184–8. doi: 10.1111/j.1365-2265.2006.02446.x, PMID: 16430718

[16] Zhong W You C Jiang S Huang S Chen H Liu J. Symptomatic Rathke cleft cyst. J Clin Neurosci. (2012) 19:501–8. doi: 10.1016/j.jocn.2011.07.022, PMID: 22336224

[17] Setian N Aguiar CH Galvão JA Crivellaro CE Dichtchekenian V Damiani D. Rathke’s cleft cyst as a cause of growth hormone deficiency and micropenis. Childs Nerv Syst. (1999) 15:271–3. doi: 10.1007/s003810050391, PMID: 10392501

[18] Langlois F Varlamov EV Fleseriu M. Hypophysitis, the growing spectrum of a rare pituitary disease. J Clin Endocrinol Metab. (2022) 107:10–28. doi: 10.1210/clinem/dgab672, PMID: 34528683 PMC8684465

[19] Eguchi K Uozumi T Arita K Kurisu K Yano T Sumida M. Pituitary function in patients with Rathke’s cleft cyst: significance of surgical management. Endocr J. (1994) 41:535–40. doi: 10.1507/endocrj.41.535, PMID: 7889113

[20] Alsavaf MB Wu KC Gosal JS Finger G Koch B Abouammo MD. Endoscopic endonasal marsupialization of rathke cleft cysts: clinical outcomes and risk factors analysis of visual impairment, pituitary dysfunction, and CSF leak. Pituitary. (2023) 26:696–707. doi: 10.1007/s11102-023-01347-y, PMID: 37878234

[21] Fan MC Wang QL Wang JF Deng WS Li LD Wang ZH. Surgical treatment of symptomatic Rathke’s cleft cysts: clinical features, therapy considerations and outcomes. Chin Med J (Engl). (2012) 125:2919–24., PMID: 22932091

[22] Menéndez-Torre EL Gutiérrez-Hurtado A Ollero MD Irigaray A Martín P Parra P. Natural history and surgical outcomes of Rathke’s cleft cysts: a Spanish multicenter study. Front Endocrinol (Lausanne). (2024) 15:1413810. doi: 10.3389/fendo.2024.1413810, PMID: 38952395 PMC11215184

[23] Trifanescu R Ansorge O Wass JA Grossman AB Karavitaki N. Rathke’s cleft cysts. Clin Endocrinol (Oxf). (2012) 76:151–60. doi: 10.1111/j.1365-2265.2011.04235.x, PMID: 21951110

[24] Zhang L Li X Li C Wang Z Zheng L Qin G. Analysis of the clinical characteristics and pituitary function of patients in central China with rathke’s cleft cysts. Front Endocrinol (Lausanne). (2022) 13:800135. doi: 10.3389/fendo.2022.800135, PMID: 35295993 PMC8919671

[25] Lin M Wedemeyer MA Bradley D Donoho DA Fredrickson VL Weiss MH. Long-term surgical outcomes following transsphenoidal surgery in patients with Rathke’s cleft cysts. J Neurosurg. (2019) 130:831–7. doi: 10.3171/2017.11.JNS171498, PMID: 29775155

[26] Langlois F Manea A Lim DST McCartney S Yedinak CG Cetas JS. High prevalence of adrenal insufficiency at diagnosis and headache recovery in surgically resected Rathke’s cleft cysts-a large retrospective single center study. Endocrine. (2019) 63:463–9. doi: 10.1007/s12020-018-1784-0, PMID: 30338480

[27] Petersson M Berinder K Eden Engström B Tsatsaris E Ekman B Wahlberg J. Natural history and surgical outcome of Rathke’s cleft cysts-A study from the Swedish Pituitary Registry. Clin Endocrinol (Oxf). (2022) 96:54–61. doi: 10.1111/cen.14622, PMID: 34724249

[28] Wait SD Garrett MP Little AS Killory BD White WL. Endocrinopathy, vision, headache, and recurrence after transsphenoidal surgery for Rathke cleft cysts. Neurosurgery. (2010) 67:837–43. doi: 10.1227/01.NEU.0000374768.16291.03, PMID: 20657318

[29] Higuchi Y Hasegawa K Kubo T Tanaka H Tsukahara H. The clinical course of Rathke’s cleft cysts in pediatric patients: impact on growth and pubertal development. Clin Pediatr Endocrinol. (2022) 31:38–43. doi: 10.1297/cpe.2021-0034, PMID: 35002067 PMC8713062

[30] Iannelli A Martini C Cosottini M Castagna M Bogazzi F Muscatello L. Rathke’s cleft cysts in children: clinical, diagnostic, and surgical features. Childs Nerv Syst. (2012) 28:297–303. doi: 10.1007/s00381-011-1626-3, PMID: 22057478

[31] Jung JE Jin J Jung MK Kwon A Chae HW Kim DH. Clinical manifestations of Rathke’s cleft cysts and their natural progression during 2 years in children and adolescents. Ann Pediatr Endocrinol Metab. (2017) 22:164–9. doi: 10.6065/apem.2017.22.3.164, PMID: 29025202 PMC5642082

[32] Lim HH Yang SW. Risk factor for pituitary dysfunction in children and adolescents with Rathke’s cleft cysts. Korean J Pediatr. (2010) 53:759–65. doi: 10.3345/kjp.2010.53.7.759, PMID: 21189952 PMC3004488

[33] Monzavi R Kelly DF Geffner ME. Rathke’s cleft cyst in two girls with precocious puberty. J Pediatr Endocrinol Metab. (2004) 17:781–5. doi: 10.1515/JPEM.2004.17.5.781, PMID: 15237714

[34] Andrysiak-Mamos E Sagan KP Zwarzany Ł Poncyljusz W Syrenicz A. The 1 μg Synacthen stimulation test in the diagnosis of secondary adrenal insufficiency in patients with Rathke’s cleft cyst and empty sella syndrome. Endokrynol Pol. (2023) 74:631–636. doi: 10.5603/ep.98271, PMID: 38159204

[35] Montaser AS Catalino MP Laws ER. Professor Rathke’s gift to neurosurgery: the cyst, its diagnosis, surgical management, and outcomes. Pituitary. (2021) 24:787–96. doi: 10.1007/s11102-021-01155-2, PMID: 34046806

[36] Oishi M Hayashi Y Sasagawa Y Kita D Tachibana O Nakada M. Outcome of diabetes insipidus in patients with Rathke’s cleft cysts. Clin Neurol Neurosurg. (2018) 167:141–6. doi: 10.1016/j.clineuro.2018.02.031, PMID: 29494890

[37] Huda S Singha S Haidous A Bukberg PR Pramanik BK Harshan M. Unraveling the mystery of rathke’s cleft cyst presenting with hyponatremia: A case report with a comprehensive review of literature. Asian J Neurosurg. (2024) 19:767–73. doi: 10.1055/s-0044-1788618, PMID: 39606298 PMC11588594

[38] Iwai H Ohno Y Hoshiro M Fujimoto M Nishimura A Kishitani Y. Syndrome of inappropriate secretion of antidiuretic hormone (SIADH) and adrenal insufficiency induced by Rathke’s cleft cyst: a case report. Endocr J. (2000) 47:393–9. doi: 10.1507/endocrj.47.393, PMID: 11075719

[39] Hsu YJ Chau T Yang SS Tsai WS Lin SH. Rathke’s cleft cyst presenting with hyponatremia and transient central diabetes insipidus. Acta Neurol Scand. (2003) 107:382–5. doi: 10.1034/j.1600-0404.2003.00036.x, PMID: 12713531

[40] Qian A Zhou J Yu J Huo G Wang X. Incidence and risk factors of delayed postoperative hyponatremia after endoscopic endonasal surgery for Rathke’s cleft cyst: A single-center study. Front Surg. (2022) 9:953802. doi: 10.3389/fsurg.2022.953802, PMID: 35910473 PMC9334746

[41] Kim JE Kim JH Kim OL Paek SH Kim DG Chi JG. Surgical treatment of symptomatic Rathke cleft cysts: clinical features and results with special attention to recurrence. J Neurosurg. (2004) 100:33–40. doi: 10.3171/jns.2004.100.1.0033, PMID: 14743909

[42] Lillehei KO Widdel L Astete CA Wierman ME Kleinschmidt-DeMasters BK Kerr JM. Transsphenoidal resection of 82 Rathke cleft cysts: limited value of alcohol cauterization in reducing recurrence rates. J Neurosurg. (2011) 114:310–7. doi: 10.3171/2010.7.JNS091793, PMID: 20799861

[43] Klein DA Paradise SL Reeder RM. Amenorrhea: A systematic approach to diagnosis and management. Am Fam Physician. (2019) 100:39–48. 31259490

[44] Current evaluation of amenorrhea: a committee opinion. Fertil Steril. (2024) 122:52–61. doi: 10.1016/j.fertnstert.2024.02.001, PMID: 38456861

[45] Febriani A WD Dwijayasa PM. Prasetyorini N case report: secondary amenorrhea with hyperprolactinemia due to pituitary macroadenoma. Asian J Heal Res. (2022) 1:46–51. doi: 10.55561/ajhr.v1i2.19

[46] Gong X Li H Zhao Y. Successful ovarian stimulation and pregnancy in an infertile woman with Rathke’s cleft cyst: a case report. Am J Transl Res. (2021) 13:13167–72., PMID: 34956537 PMC8661215

[47] Andrysiak-Mamos E Sagan K Sagan L Sowińska-Przepiera E Syrenicz A. Cystic lesions of the sellar-suprasellar region - diagnosis and treatment. Endokrynol Pol. (2018) 69:212–28. doi: 10.5603/EP.2018.0023, PMID: 29952427

[48] Cote DJ Iuliano SL Catalino MP Laws ER. Optimizing pre-, intra-, and postoperative management of patients with sellar pathology undergoing transsphenoidal surgery. Neurosurg Focus. (2020) 48:E2. doi: 10.3171/2020.3.FOCUS2043, PMID: 32480374

[49] Freda PU Beckers AM Katznelson L Molitch ME Montori VM Post KD. Pituitary incidentaloma: an endocrine society clinical practice guideline. J Clin Endocrinol Metab. (2011) 96:894–904. doi: 10.1210/jc.2010-1048, PMID: 21474686 PMC5393422

[50] Fleseriu M Hashim IA Karavitaki N Melmed S Murad MH Salvatori R. Hormonal replacement in hypopituitarism in adults: an endocrine society clinical practice guideline. J Clin Endocrinol Metab. (2016) 101:3888–921. doi: 10.1210/jc.2016-2118, PMID: 27736313

[51] Molitch ME Clemmons DR Malozowski S Merriam GR Vance ML. Evaluation and treatment of adult growth hormone deficiency: an Endocrine Society clinical practice guideline. J Clin Endocrinol Metab. (2011) 96:1587–609. doi: 10.1210/jc.2011-0179, PMID: 21602453

[52] Aydin S Darko K Detchou D Barrie U. Rathke’s cleft cysts: from pathophysiology to management. Neurosurg Rev. (2024) 47:522. doi: 10.1007/s10143-024-02742-0, PMID: 39223314

[53] Karavitaki N Scheithauer BW Watt J Ansorge O Moschopoulos M Llaguno AV. Collision lesions of the sella: co-existence of craniopharyngioma with gonadotroph adenoma and of Rathke’s cleft cyst with corticotroph adenoma. Pituitary. (2008) 11:317–23. doi: 10.1007/s11102-007-0070-6, PMID: 17917812

[54] Sagan KP Andrysiak-Mamos E Sagan L Nowacki P Małkowski B Syrenicz A. Cushing’s syndrome in a patient with rathke’s cleft cyst and ACTH cell hyperplasia detected by (11)C-methionine PET imaging-A case presentation. Front Endocrinol (Lausanne). (2020) 11:460. doi: 10.3389/fendo.2020.00460, PMID: 32774326 PMC7388627

[55] Park M Lee SK Choi J Kim SH Kim SH Shin NY. Differentiation between cystic pituitary adenomas and rathke cleft cysts: A diagnostic model using MRI. AJNR Am J Neuroradiol. (2015) 36:1866–73. doi: 10.3174/ajnr.A4387, PMID: 26251436 PMC7965051

[56] Babu R Back AG Komisarow JM Owens TR Cummings TJ Britz GW. Symptomatic Rathke’s cleft cyst with a co-existing pituitary tumor; Brief review of the literature. Asian J Neurosurg. (2013) 8:183–7. doi: 10.4103/1793-5482.125662, PMID: 24551002 PMC3912769

[57] Candy NG Mignone E Quick E Koszyca B Brown A Chapman IM. The role of BRAF testing of Rathke’s cleft cysts to identify missed papillary craniopharyngioma. Pituitary. (2025) 28:30. doi: 10.1007/s11102-025-01501-8, PMID: 39900703 PMC11790742

[58] Zheng Y Foo JQX Xu X Nga VDW. Surgical management of symptomatic recurrent Rathke’s cleft cysts: A systematic review and individual-participant data meta-analysis. J Clin Neurosci. (2024) 130:110917. doi: 10.1016/j.jocn.2024.110917, PMID: 39541655

[59] Agarwalla PK Koch MJ Royce TJ Redjal N Bussière MR Loeffler JS. Stereotactic radiation as salvage therapy for recurrent rathke cleft cysts. Neurosurgery. (2020) 87:754–60. doi: 10.1093/neuros/nyz523, PMID: 31942633

[60] Giraldi E Allen JW Ioachimescu AG. Pituitary incidentalomas: best practices and looking ahead. Endocr Pract. (2023) 29:60–8. doi: 10.1016/j.eprac.2022.10.004, PMID: 36270609

[61] Qian A Zhou J Zhang X Yu J Wang X. Incidence and factors associated with the recurrence of Rathke’s cleft cyst after surgery: A systematic review and meta-analysis. Front Surg. (2022) 9:1065316. doi: 10.3389/fsurg.2022.1065316, PMID: 36684167 PMC9849585

[62] el-Mahdy W Powell M. Transsphenoidal management of 28 symptomatic Rathke’s cleft cysts, with special reference to visual and hormonal recovery. Neurosurgery. (1998) 42:7–16. doi: 10.1097/00006123-199801000-00003, PMID: 9442498

[63] Mendelson ZS Husain Q Elmoursi S Svider PF Eloy JA Liu JK. Rathke’s cleft cyst recurrence after transsphenoidal surgery: a meta-analysis of 1151 cases. J Clin Neurosci. (2014) 21:378–85. doi: 10.1016/j.jocn.2013.07.008, PMID: 24269553

[64] Cabuk B Selek A Emengen A Anik I Canturk Z Ceylan S. Clinicopathologic characteristics and endoscopic surgical outcomes of symptomatic Rathke’s cleft cysts. World Neurosurg. (2019) 132:e208–e16. doi: 10.1016/j.wneu.2019.08.196, PMID: 31493602

[65] Nakase K Nishimura F Morisaki Y Yokoyama S Kotsugi M Takeshima Y. High recurrence of Rathke’s cleft cysts with anterior-Inferior pituitary displacement despite standard surgical approaches. Pituitary. (2025) 28:82. doi: 10.1007/s11102-025-01554-9, PMID: 40593326

[66] Oyama N Tahara S Oyama K Ishii Y Teramoto A. Assessment of pre- and postoperative endocrine function in 94 patients with Rathke’s cleft cyst. Endocr J. (2013) 60:207–13. doi: 10.1507/endocrj.EJ12-0039, PMID: 23171703

[67] Sonnet E Roudaut N Mériot P Besson G Kerlan V. Hypophysitis associated with a ruptured Rathke’s cleft cyst in a woman, during pregnancy. J Endocrinol Invest. (2006) 29:353–7. doi: 10.1007/BF03344108, PMID: 16699303

[68] Zada G. Rathke cleft cysts: a review of clinical and surgical management. Neurosurg Focus. (2011) 31:E1. doi: 10.3171/2011.5.FOCUS1183, PMID: 21721866

[69] Iqbal J Kanaan I Al Homsi M. Non-neoplastic cystic lesions of the sellar region presentation, diagnosis and management of eight cases and review of the literature. Acta Neurochir (Wien). (1999) 141:389–97. doi: 10.1007/s007010050315, PMID: 10352749

[70] Refardt J Atila c Christ-Crain M. New insights on diagnosis and treatment of AVP deficiency. Rev Endocr Metab Disord. (2024) 25(3):639–49. doi: 10.1007/s11154-023-09862-w, PMID: 38087160 PMC11162367

